# Thoracic compartment syndrome after penetrating heart and lung injury

**DOI:** 10.3205/iprs000133

**Published:** 2019-04-04

**Authors:** Holger Rupprecht, Harald Dormann, Katharina Gaab

**Affiliations:** 1 Surgical Department 1, Clinical Center Fürth, Germany; 2 Emergency Department, Clinical Center Fürth, Germany

**Keywords:** penetrating heart and lung injury, compartment syndrome, myocardial and lung edema, packing, bogota bag

## Abstract

Thoracic injuries are the most lethal penetrating injuries.

After attempting suicide, two patients with a penetrating thoracic wound were admitted to our emergency department. During CT scan they became hemodynamically unstable, which is why we had to perform an emergency thoracotomy.

In both cases, a perforation in the left ventricle as well as multiple lesions of the lung parenchyma and vessel injuries were found. After the treatment of the different injuries, a massive edema of the heart and lung prevented a primary closure of the thorax. Due to massive diffuse bleeding, a “packing“ of the pleural cavity became necessary.

To prevent a thoracic compartment syndrome, the thoracic wall was left open and the skin was closed with a plastic sheet.

Due to the “open chest” procedure combined with “packing” of the thoracic cavity, the majority of patients with an edema of the heart and lung after a penetrating chest injury can be saved. Pitfalls of preclinical and clinical treatment, aspects of diagnostics and surgery are discussed.

## Zusammenfassung

Thorakale Verletzungen sind die tödlichsten penetrierenden Verletzungen.

Zwei Patienten wurden nach einem Suizidversuch mit einer penetrierenden Thoraxverletzung in unsere Notaufnahme eingeliefert. Während der CT-Untersuchung kam es zu einer Kreislaufdekompensation, so dass die notfallmäßige Thorakotomie erfolgen musste.

In beiden Fällen fand sich eine Perforation im linken Ventrikel neben multiplen Lungenparenchym- und Gefäßverletzungen. Nach Versorgung der verschiedenen Läsionen verhinderte ein massives Ödem des Herzens und der Lunge einen Primärverschluss des Thorax, wobei wegen diffuser Blutung noch ein „Packing“ der Thoraxhöhle notwendig wurde. Zur Prävention eines Kompartmentsyndroms blieb der knöcherne Thorax offen und es wurde nur die Haut mit einer Plastikfolie verschlossen.

Aufgrund der Kombination des offen belassenen Thorax und des Austamponierens der Thoraxhöhle kann die Mehrzahl der Patienten mit einem Herz-Lungen-Ödem nach penetrierender Verletzung gerettet werden.

Fallstricke der präklinischen und klinischen Behandlung sowie Aspekte der Diagnostik und Operation werden diskutiert.

## Introduction

Most patients with blunt and penetrating chest trauma die at the accident site [1]. Knowledge of the mechanism of injury, precise clinical anamnesis and examination as well as the appropriate use of diagnostic tools will help to choose the right surgical access and to realize the lethal complications related to cardiac injury.

## Case descriptions

**Figure 1:**
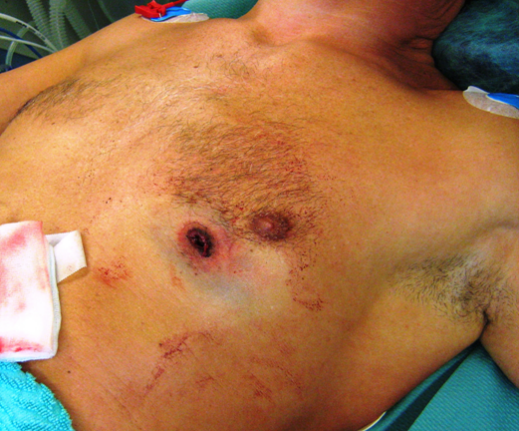
Entry wound of 45-Magnum-revolver

**Figure 2:**
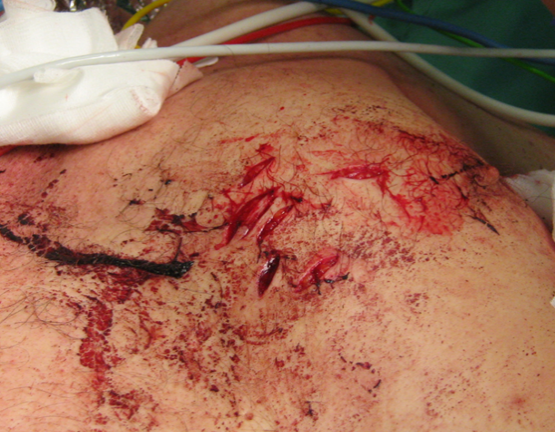
Thoracic wound of the letter opener

**Figure 3:**
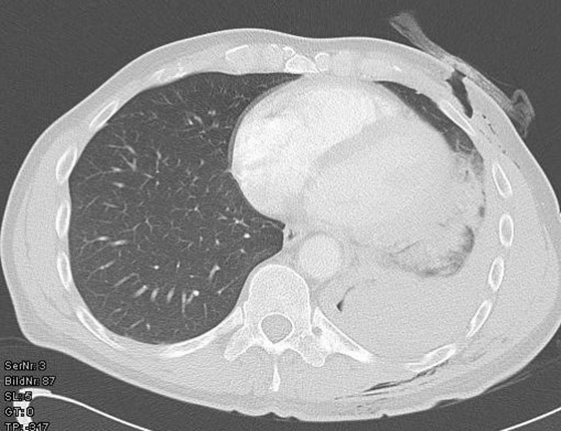
Computer-assisted tomography: hematothorax

**Figure 4:**
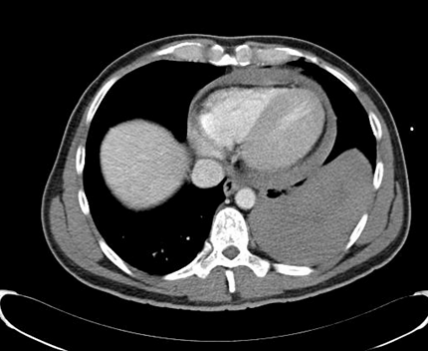
Computer-assisted tomography: pericardial tamponade

Two patients (42- and 49-year-old) were admitted to our emergency department after a suicide attempt. In the first case, the patient stabbed himself in the left chest with a letter opener. In the second case, the 49-year-old man shot himself with a 45-magnum-revolver into the anterior left chest wall. The clinical examination showed rarely bleeding thoracic wounds (Figure 1 (Fig. 1), Figure 2 (Fig. 2)). The patients were responsive, completely oriented (GCS=15) and hemodynamically stable. The abdominal sonography (FAST) showed no free fluid. Due to unclear injury patterns, a CT scan was performed. Shortly after the completion of the investigation, the patients became hemodynamically unstable with upper venous congestion. The patients were immediately transported to the operating room. In both cases, the computed-assisted tomography verified a massive left hematothorax and a pericardial tamponade (Figure 3 (Fig. 3), Figure 4 (Fig. 4)). In the case of the 42-year-old patient, a sternotomy was performed initially, followed by an anterolateral thoracotomy to supply the intercostal vessels more sufficiently. The second patient was treated with a left anterolateral thoracotomy. In both cases, a blood-filled pleural cavity, a lesion of the left ventricle as due to the pericardial tamponade and extracardiac injuries were found (Table 1 (Tab. 1)). 

Due to the massive bleeding, the situs was initially unclear. Therefore, the lung hilum was primarily disconnected for bleeding control and after the continuous suction of the pleural cavity with the cell saver, the specific disconnection of the ruptured parts of the lung was performed.

The damaged vessels were supplied with non-absorbable sutures and the destroyed lung parenchyma was resected atypically using a stapler devise. The perforation channel of the lung perforations was treated by tractotomy with the help of a linear cutter. The bronchial tubes and vessels were supplied with sufficient seam [2]. 

Case 1: After the pericardiotomy and the evacuation of the blood clots, a “chemical asystole” was induced through the administration of adenosine (Adrekar^®^) to close the lesion in the left ventricle more easily. 

**Figure 5:**
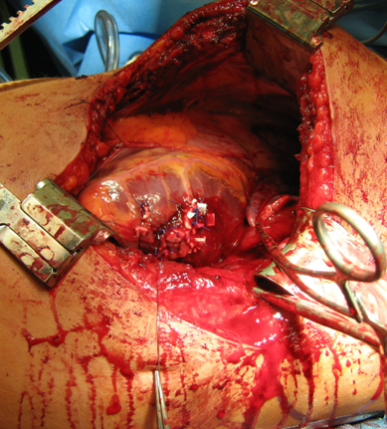
Left ventricle with non-absorbable sutures sheathed with teflon plates (Prolene 2-0)

**Figure 6:**
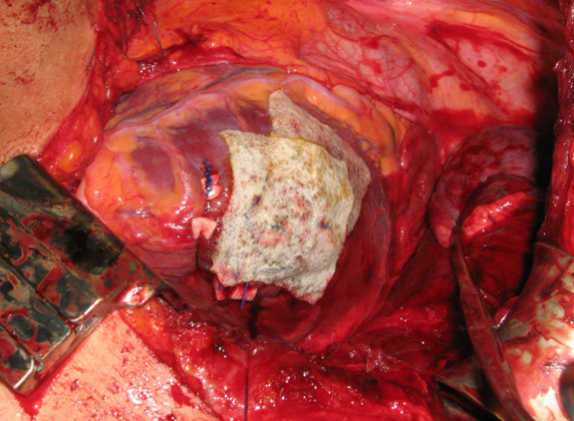
Left ventricle sealed with fibrin fleeces (Tachosil^®^)

Case 2: The blast wave of the projectile led to a myocardial contusion with secondary delayed rupture of the left ventricle. Initially, the massive bleeding from the 2 cm intraventricular defect was only manageable through the insertion of a blocked Fogarty catheter. With non-absorbable sutures sheathed with teflon plates (Prolene 2-0) the defect was closed and the leackage of the stitches was sealed sufficiently with two fibrin fleeces (Tachosil^®^) (Figure 5 (Fig. 5), Figure 6 (Fig. 6)).

**Figure 7:**
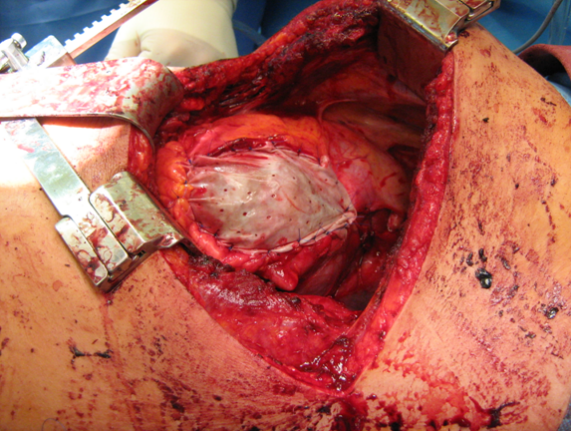
Pericardium with bovine patch (Tutomesh^®^)

The shock related ischemia led to a massive myocardial and non-cardiac lung edema. A closure of the pericardium was not possible as otherwise it would have resulted in an “iatrogenic” tamponade. Because of that, an “extension plastic” with a Vicryl mesh (Case 1) and a bovine patch (Tutomesh^®^) (Case 2) was sewed into the pericardium (Figure 7 (Fig. 7)).

**Figure 8:**
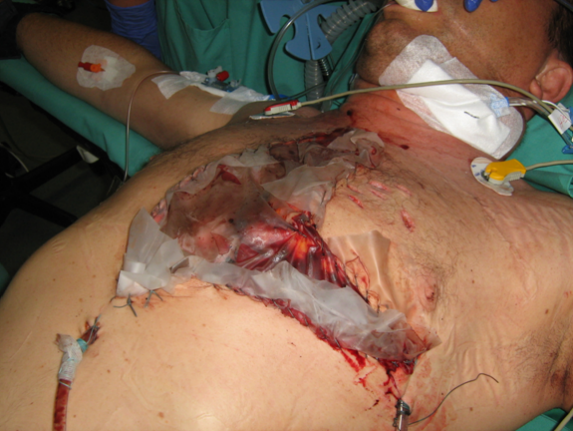
Thoracic “Bogota bag”

The massive swollen lung also prevented a primary closure of the thorax. The first attempt resulted in a systemic drop in blood pressure and a massive increase of the ventilation pressure. At this time, a so-called “lethal triad” was present (acidosis, hypothermia, coagulopathy). The result was a diffuse bleeding, which was only manageable through “packing” of the pleural cavity. As a consequence, the “expansion room” of the lung was reduced more and the thoracic wall was left open. An ordinary plastic sheet which is sewed into the skin (Figure 8 (Fig. 8)) helps the edematous lung to expand. After stabilization on the intensive care unit (normothermia, correction of the acid-base equilibrium), the tamponades could be removed and the thorax was closed definitely after 24 hours. The wounds healed per primam under antibiotic covering. After ambulant psychiatric care, the patients are fully restored without physically afflictions.

## Discussion

Patients with a pericardial tamponade can stay hemodynamically stable for a longer time. Clinical symptoms vary or can be completely missing until the intrapericardial pressure exceeds the diastolic pressure, which leads to a circulatory collapse [3], [4], [5]. Because of that, penetrating thoracic traumata must be considered as “worst case scenarios” until the contrary is proven. Aggressive diagnostics and therapy are required. The “time factor” plays an important role for prognosis [5], [6], [7], [8]. 

In the cases of our patients, two fatal mistakes could have led to a lethal outcome. Both patients were transported seated by private persons. Normally, an emergency team has to be alerted. Unfortunately, not in all cases a preclinical big lumen thoracic drainage (28 Ch.) is inserted, although in most of the cases additional intrathoracic injuries are present [9]. Especially in cases of a long transportation time, it is fatal not to place a thoracic drainage on the injured site. Most patients with this injury pattern die of a tension pneumothorax, for example, on the way to the hospital. They do not primarily die of a myocardial lesion [5].

The second “pitfall” concerns the clinical diagnostics in the trauma room. The detection of a pericardial hematoma during FAST of the pericarium and the thorax is enough for emergency thoracotomy in cases of a penetrating thoracic trauma. The detection of hematothorax indicates immediate thoracic drainage [3], [10], [11].

In cases of an acute blood loss of 1,000 ml over the drainage or continuous blood loss of 200–300 ml over 3 hours, an emergency thoracotomy is necessary [5], [12], [13].

A primary hemodynamical stable patient and a normal ECG (electrocardiogram) can create a false sense of security [3], [6]. 

If the stabilization of the patient is not possible after the rapid infusion of 2 liters [6], [11] or a cardiopulmonal reanimation has already become necessary, a trauma-room thoracotomy should be performed [2], [14], [15]. 

Standard access is the lateral thoracotomy, which allows the disconnection of the lung hilum (also for prevention of an air embolism), the pericardiotomy and the direct heart massage [4], [5], [6], [7], [8], [13], [16], [17]. The left lateral thoracotomy also allows the disconnection of the descending aorta over the diaphragm to improve the blood circulation of the coronary arteries retrogradely [4].

The “clemshell thoracotomy” (transverse sternotomy) or the median sternotomy allows the inspection of the mediastinum and of both pleural cavities [4], [7], [8], [16].

In cases of emergency thoracotomy in the trauma roomm the clemshell technique should be preferred due to of an easier handling. After opening the thorax, the lung hilum should be disconnected [4], [17] to detect the reason for the bleeding after the suction with the cell saver [5], [8]. A longer central disconnection may lead to acute right heart failure [18]. After pericardiotomy, smaller perforations in the heart wall should be manually compressed and can be sewed with non-absorbable stitches [4], [5], [19].

Bigger defects can be occluded primarily with the help of a Fogarty or urinary catheter to prevent bleeding to death [4], [16], [20]. The urinary catheter also allows an intracardial fluid resuscitation [16]. With a 16 Charrière urinary catheter up to 250 ml/minute can be administrated. The intravenous administration of adenosine (Adrekar^®^: 0,15–0,30 mg/kg KG) leads, through to a short block of the AV (atrioventricular) node, to an asystole, which simplifies the treatment of heart injuries [19], [21].

The shock related ischemia and hypothermia lead to a massive edema of the myocardium and the lung parenchyma [7], [11], [17], [18], [22] and to a coagulation disorder. The massive diffuse bleeding is not manageable with transfusion of fresh frozen plasma (FFP) and blood reserves alone [23]. Only the tamponade of the pleural cavity (“packing”) is able to prevent a lethal outcome [11], [17], [23], [24]. This method which was first used in cases of massive intraabdominal bleeding (e.g. rupture of the liver) [25], [26] was later used in thoracic surgeries [7], [18], [27] and after successful application in cardiac surgeries [28], [29]. The tamponade of the pleural cavity can impede the diastolic filling of the heart as well as the expansion of the lung [11], [18], [27]. This can be prevented through the “open chest” procedure [4], [11], [18], [23], [30], [31]. A massive non-cardiac edema of the lung leads to a thoracic compartment syndrome in case of primary thoracic closure [7], [11], [17], [18], [31], [32]. Warning signs can be an increase in ventilation pressure and a drop of blood pressure [7], [11], [32], which can lead to cardiac arrest. To prevent this fatal complication, the thoracic wall [7], [11], [31], [32] and muscles are left open and only an ordinary plastic sheet should be sewed into the skin (modified “Bogota bag”). This easy, cheap and always applicable method has proven its worth in septic abdominal surgery [33]. Through expansion of the pleural cavity, even a massive edematous lung is able to expand after packing without affecting intrathoracic organs [23].

Furthermore, due to the myocardial edema the pericardium cannot be closed during shock. Trying to primarily close the pericardium under tension leads to an iatrogenic tamponade with a drop of the cardiac output.

Through a pericardial patch plastic with bovine pericardium (Tutomesh^®^) or with alloplastic materials (Goretex^®^), this complication can be avoided [5], [34].

After “damage control” the patient should be stabilized on the ICU (intensive care unit) for example through the regulation of temperature and correction of the acid-base-balance [35], [36]. Cardiac diagnostics such as echocardography (transthoracal or transesophageal) should be performed in this phase to exclude intracardial lesions, e.g. ventricular septal defect [14], [37].

Usually 24–36 hours after stabilization, the tamponades can be removed and the thorax can be closed after the insertion of big lumen thoracic drainages [38].

## Conclusion

In cases of penetrating heart and lung injuries, not only a massive edema of the intrathoracic organs [17], [18] results due to the shock but also a massive coagulation disorder with consecutive diffuse bleeding is visible. 

This primarily leads to a thoracic compartment syndrome [7], [11], [32] and at last to a cardiac arrest and bleeding to death. The massive diffuse bleeding is not manageable with transfusion of fresh frozen plasma (FFP) and blood reserves alone. Only the ”packing” of the pleural cavity [23], [24] and the “open chest” procedure [5], [11], [31] with a so called “Bogota bag” [33] can save the life of patients with such injury patterns.

## Notes

### Competing interests

The authors declare that they have no competing interests.

## Figures and Tables

**Table 1:**
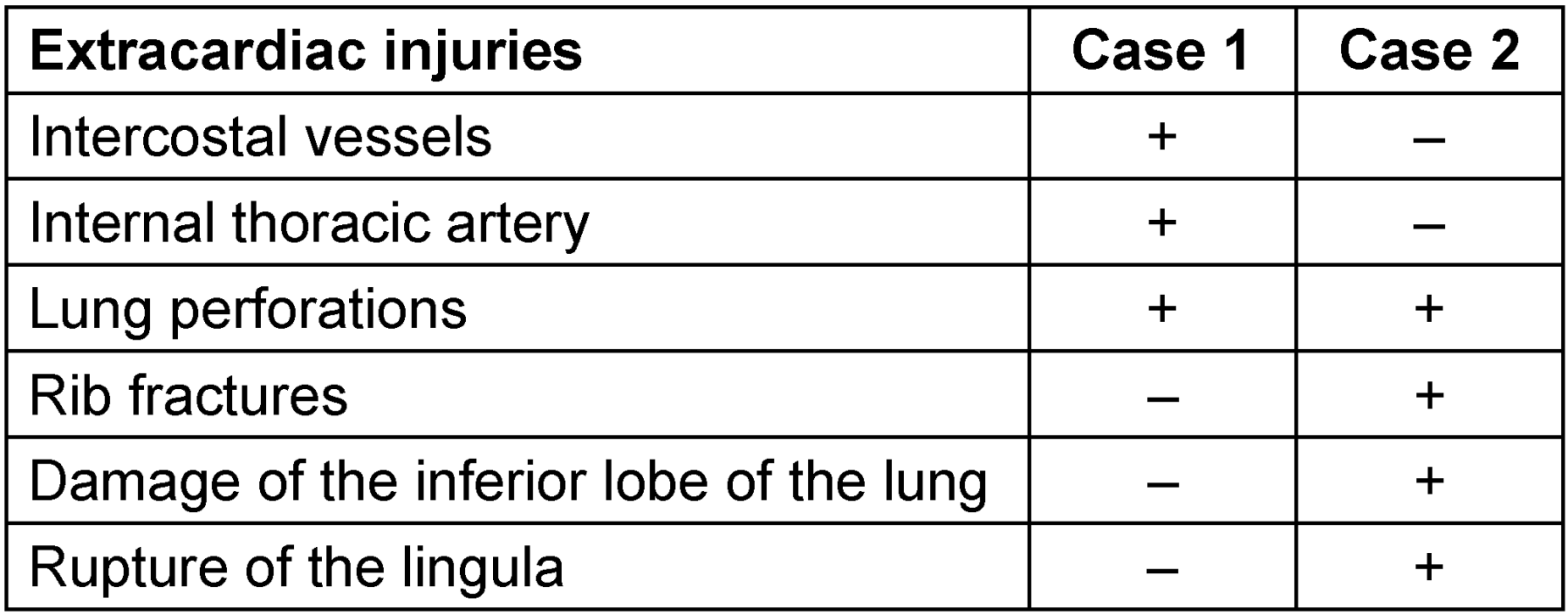
Extracardiac injuries
